# The pathological characteristics of enzootic nasal adenocarcinoma in goats

**DOI:** 10.2478/jvetres-2025-0010

**Published:** 2025-03-01

**Authors:** Lingxu Li, Zhen Wang, Weiling Qi, Yingjun Lv, Dawei Yao

**Affiliations:** College of Veterinary Medicine, Nanjing Agricultural University, Nanjing, 210095, China

**Keywords:** enzootic nasal adenocarcinoma, enzootic nasal tumour virus, goat, immunohistochemistry, ultrastructure

## Abstract

**Introduction:**

Enzootic nasal adenocarcinoma (ENA) is a nasal cancer that occurs in goats and sheep infected by enzootic nasal tumour virus. Pathologic examinations are useful for distinguishing tumours from inflammatory hyperplasia. The aim of this study was to describe the pathological characteristics of ENA.

**Material and Methods:**

Caprine tumour samples were collected for pathological examination. The tissue sections were stained with haematoxylin-eosin and periodic acid-Schiff (PAS) and processed for immunohistochemical staining. Tumour samples were also processed for routine transmission electron microscopy (TEM).

**Results:**

The histopathological structure of the tumours exhibited both papillary formations in the superficial regions and tubular or acinar formations in the deeper layers, representing distinct structural patterns within the same adenocarcinoma. The tumour cells were positive for PAS, and mitotic figures were rare. Low-differentiated cancer nests and epithelial–mesenchymal transition phenomena were observed. Immunohistochemical analysis showed that the tumour cells were strongly positive for pancytokeratin and cytokeratin (CK)18, moderately positive for CK7, and did not express olfactory marker protein. The Kiel 67 labelling index was approximately 23%. Retrovirus-like particles were distributed inside and outside of acinar tumour cells in TEM.

**Conclusion:**

The origin site of ENA is the epithelium of the nasal glandular tubules. This cancer is a low-grade adenocarcinoma with malignant potential. Cytokeratin 7 and CK18 can be considered immunophenotypes for identifying ENA tumour cells.

## Introduction

Enzootic nasal adenocarcinoma (ENA) is a specific nasal cancer that occurs in goats and sheep (15, 23). In China, there are no reports of ENA in sheep, but ENA in goats has been reported in several provinces and has been occurring over more of the country in recent years (11). Clinical signs in affected goats are similar to those in other respiratory diseases, and pathological examination is an important method of distinguishing tumours from inflammatory hyperplasia (2). Many case reports of ENA have shown histopathological tumour images (18, 21). Initially, researchers thought that the tumour cells originated from the olfactory mucosa Bowman’s glands in the ethmoid region, but a later immunohistochemical study using lysozyme staining found that the tumour cells’ staining pattern was the same as that of the serous glands (17). Currently, ENA tumours are classified as low-grade adenocarcinoma, and it is believed that ENA does not metastasise to other organs (4, 20). In many case reports, retrovirus-like virus particles were detected in tumour tissue under transmission electron microscopy (TEM), which also provided evidence for tumour formation by enzootic nasal tumour virus (ENTV) infection in goats (5).

In order to further characterise the gross, histopathological, immunohistochemical and ultrastructural characteristics of ENA, tumour tissues were collected from five dead goats with the adenocarcinoma. Periodic acid–Schiff (PAS) and haematoxylin-eosin (HE) staining were performed to differentiate and observe the morphological characteristics differentiate of tumour cells. Pancytokeratin (Pan-CK), cytokeratin 7 (CK7), cytokeratin 18 (CK18), olfactory marker protein (OMP) and Kiel 67 were examined in ENA tumours to clarify the immunohistochemical characteristics of ENA, analyse the tumour origin and screen for suitable antibodies for ENA tumour cell identification. In addition, the ultrastructure of tumour cells was observed in TEM to determine the cell characteristics and viral particle distribution within it.

## Material and Methods

### Samples

Five goats of the indigenous Chinese Anhui white breed, aged 0.8–2 years, from the same farm and commingled, became sick on a goat farm during the study period. The clinical manifestations of their disease included heavy respiratory sounds, unilateral or bilateral serous nasal discharge and reddening of the skin around the nostrils. Loss of appetite, emaciation, weaker rumination, depression and reluctance to stand were some of the clinical signs of the goats with more severe respiratory symptoms. Enzootic nasal tumour virus 2 (ENTV-2) was detected by PCR in the nasal secretion samples collected from five goats (9). The five diseased goats were dissected immediately after death. The nasal cavity was exposed by cutting the goat’s head along the median sagittal plane with a bone saw. Tumour samples were collected for pathological examination.

### Histopathology

Tumour samples were fixed in 10% neutral buffered formalin solution and embedded in paraffin. The tissue sections were stained with HE and PAS and observed under an optical microscope.

### Immunohistochemistry

Sections were then processed for immunohistochemical staining, which was achieved with anti-Pan-CK (1 : 500; Mouse Monoclonal Antibody TPB-0029; Typing Biotech Co., Nanjing, China), anti-CK7 (1 : 200; Rabbit Monoclonal Antibody DF7027; Affinity Biosciences, Cincinnati, OH, USA), anti-CKl8 (1 : 200; Rabbit Monoclonal Antibody AF0191; Affinity Biosciences), anti-OMP (1 : 500; Rabbit Monoclonal Antibody ab183947, abcam, Cambridge, UK) and anti-Kiel 67 (1 : 500; Rabbit Monoclonal Antibody TPB-0067; Typing Biotech Co.). Ethmoidal labyrinth samples from healthy Anhui white goats were used as controls and samples without the addition of primary antibody were used as negative controls. Immunohistochemical examination sections were routinely processed according to the antibody manufacturers’ instructions.

All sample sections were digitally scanned using a Pannoramic MIDI (3DHistech, Budapest, Hungary) and evaluated with Slide Viewer software v. 2.5 (3DHistech). Kiel 67-positive cells were counted using the NuclearQuant built-in image analysis application (3DHistech), which can quantify both the frequency and intensity of nuclear staining. Nuclei were appraised and given one of four different scores: 0 signified no staining, 1+ reflected low-intensity, 2+ moderate-intensity and 3+ high-intensity staining. All nuclei in the section were counted and given a graded score, and the number of nuclei rated as 3+ was divided by the total number of nuclei. The ratio of the Kiel 67-positive cells to tumour cell nuclei was found.

### Ultrastructural pathology

Tumour samples were also processed for routine TEM examination. The intranasal tumour was gently washed 2 or 3 times with sterile phosphate buffered saline (PBS, pH 7.2) to remove the mucus on the surface of the tumours. The tumour samples were cut to a size of 5 mm × 2 mm × 1 mm with a double-sided blade, fixed with 2.5% glutaraldehyde, dehydrated in graded concentrations of ethanol and embedded in epoxy resin. Thin sections cut with diamond knives were placed on copper grids and injected with uranyl acetate and lead citrate. The ultrathin sections were observed with a TEM (H-7650; Hitachi, Tokyo, Japan).

## Results

### Autopsy examination

**Fig. 1.**
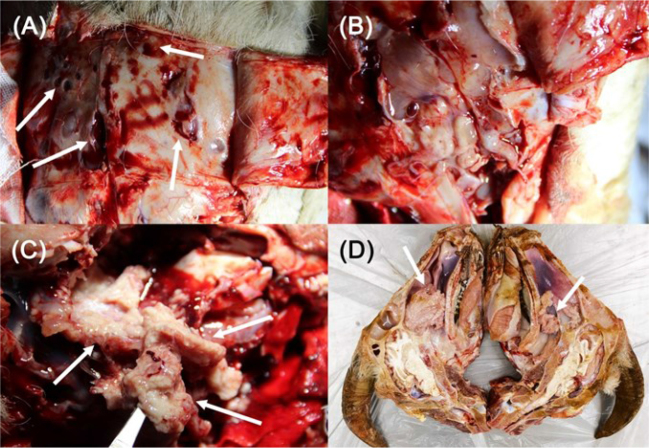
Dissection pictures of the nasal cavity in Chinese Anhui white goats with enzootic nasal adenocarcinoma. A – varying degrees of osteolysis (white arrows) in the frontal bone; B – tumour surface covered with a large amount of mucus; C – pale white and cauliflower-like tumours with a granular surface (white arrows); D – goat’s head incised along the paramedian sagittal plane exposing a large tumour (white arrow) deep in the nasal cavity obstructing the nasopharynx

After incision and reflection of the frontal facial skin of the dead goats, the surface of the frontal bone was observed to be irregular with rounded foci of osteolysis (Fig. 1A). Removal of the frontal bone from the frontal sinus surface revealed that the frontal sinuses were filled with mucoid discharge (Fig. 1B). The five goats each had either unilateral (two) or bilateral (three) tumours in the nasal cavity. The tumours were pale white or pink and cauliflower-like, with a large amount of mucus covering the surface (Fig. 1C). The lesion was closely connected with the ethmoidal labyrinth and extended to the front of the nasal cavity along the turbinate bone (Fig. 1D). The ethmoid and turbinate bones showed varying degrees of destruction and displacement correlated with tumour size. The tumours had invaded the nasopharynx in the goats with severe clinical symptoms, which caused choanae obstruction (Fig. 1D). The affected portions of the ethmoid and turbinate bones were embedded in the tumour masses. No significant changes were observed in other tissues or organs.

### Histopathological characteristics

**Fig. 2.**
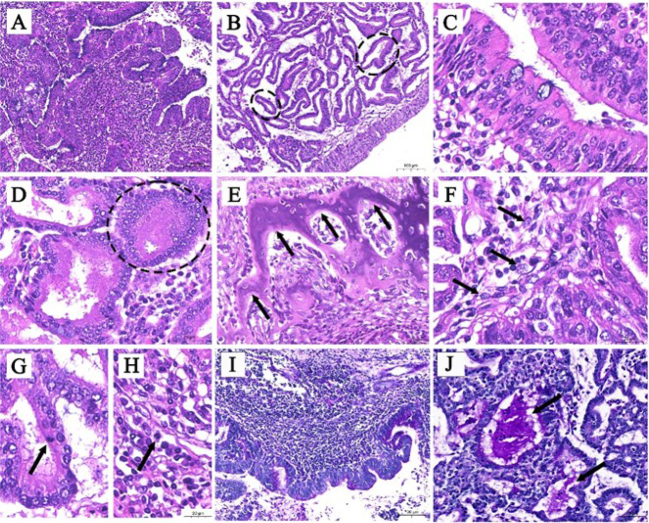
Representative haematoxylin-eosin and periodic acid–Schiff staining pictures of a caprine enzootic nasal adenocarcinoma. A – papillae located in the superficial layer of the tumour; B – acini located in the deeper layer of the tumour (broken black lines); C – papillae covered with a pseudostratified ciliated columnar epithelium; D – acinus consisting of a cuboidal epithelium of two to four layers (broken black line); E – remnant hyaline cartilage within the tumour tissue, with structure no longer intact (black arrows); F – epithelial-mesenchymal transition (black arrows) of tumour cells; G and H – mitotic figures (black arrow) of acinar and stromal tumour cells; I – a large number of PAS-positive granules on the cell surface; J – uneven, clumped purple granules aggregated in the acinar lumens (black arrows)

The caprine tumours were adenocarcinomas with predominantly well-differentiated and less frequently poorly differentiated regions. In histopathological sections, the tumour cells were organised into two structural patterns within the same adenocarcinoma: papillary formations in the superficial layer (Fig. 2A) and acinar formations in the deeper layer (Fig. 2B). The papillae were finger-like overall and were covered with a pseudostratified ciliated columnar epithelium. Tumour cells had large, rounded nuclei and thickened nuclear membranes. The nucleus was unevenly stained, pale and usually in the basal position (Fig. 2C). There was no significant change in the number and shape of goblet cells (Fig. 2C), which were interspersed among the epithelial cells. The stroma inside the papillary region was composed of connective tissues, including a large number of lymphocytes, macrophages, plasma cells and fibroblasts.

The internal tumour cells were arranged in acinar patterns. Most of the acinus consisted of a single layer of columnar or cuboidal epithelium, and the remainder was cuboidal epithelium of two to four layers (Fig. 2D). The borders of the acinar tumour cells were distinct, and the cytoplasm was eosinophilic or neutrophilic. The nuclei of acinar tumour cells were enlarged, round or oval, and located on one side of the cytoplasm or in the centre. The stroma between acini was loose connective tissue with arterioles and venules. There was no apparent tumour infiltration in the vessels and no apparent increase in lymphoid follicles. There was also remnant hyaline cartilage within the tumour tissue (Fig. 2E), probably ethmoid or turbinate bone. The normal bone structure was lost and its edge was crushed and eroded by the tumours.

Distinct epithelial–mesenchymal transition (EMT) was observed at the junction of acinar tumour cells and stromal cells in all affected goats. The epithelial cells began the morphological transition into elongated fibre cells (Fig. 2F). Mitotic figures of acinar (Fig. 2G) and papillary (Fig. 2H) tumour cells were rarely observed, one or none being noted per 10 high-power fields.

The PAS-positive granules were distributed in both the acini and papillae. There were more purple granules in pseudostratified ciliated columnar epithelia than there were on the papillae (Fig. 2I). No significant change was observed in the relative number or staining strength of goblet cells. The cytoplasm of the acinar cells was widely distributed with obvious and heterogeneous PAS-positive granules. Uneven, clump-like purple granules were aggregated in some acinar lumens (Fig. 2J).

### Immunohistochemical characteristics

**Fig. 3.**
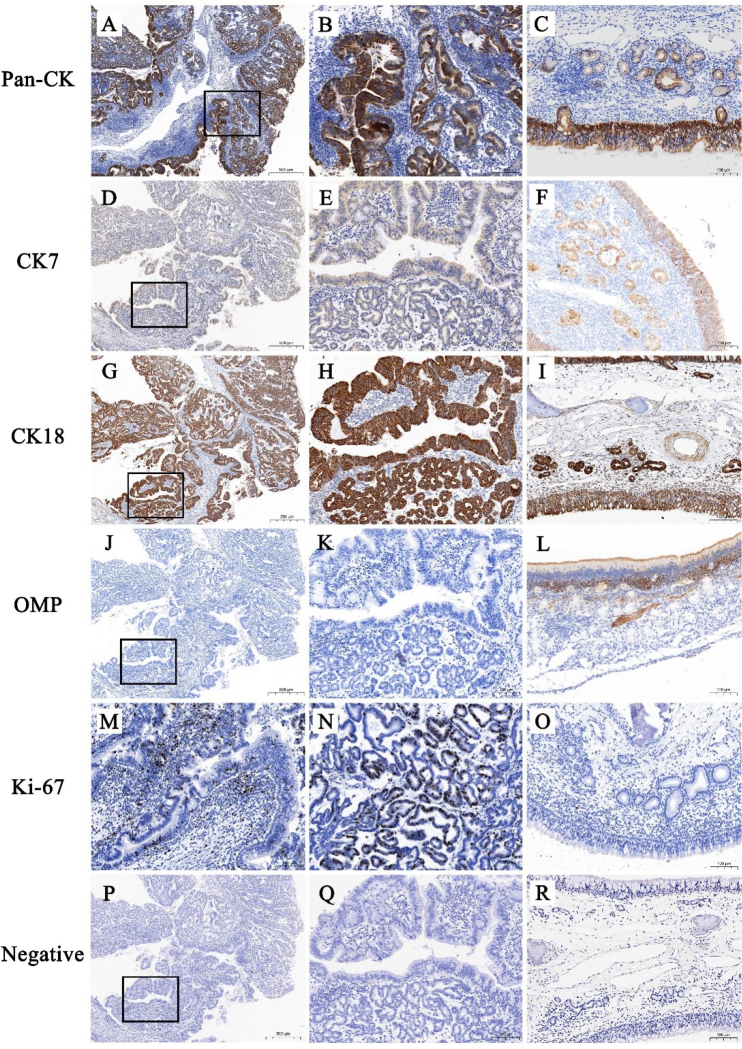
Representative immunohistochemical pictures of enzootic nasal adenocarcinomatous and healthy goat ethmoidal labyrinth. A and B – strong, diffuse cytoplasmic expression of pancytokeratin (Pan-CK) in a tumour epithelium; C – positive cytoplasmic expression of Pan-CK in a normal ethmoidal labyrinth epithelium; D and E – moderate, diffuse cytoplasmic expression of cytokeratin 7 (CK7) in a tumour epithelium; F – positive cytoplasmic expression of CK7 in a normal ethmoidal labyrinth epithelium; G and H – strong, diffuse cytoplasmic expression of cytokeratin 18 (CK18) in a tumour epithelium; I – positive cytoplasmic expression of CK18 in a normal ethmoidal labyrinth epithelium; J and K – no expression of olfactory marker protein (OMP) in a tumour epithelium; L – positive cytoplasmic expression of OMP in a normal ethmoidal labyrinth epithelium; M and N – Kiel 67-positive nuclei in a tumour epithelium with a Kiel 67 index of approximately 23%; O – near absence of Kiel 67-positive nuclei in a normal ethmoidal labyrinth epithelium with a Kiel 67 index of close to 0%; P, Q and R – negative control for tumour tissue and a normal ethmoidal labyrinth. Black boxes (A, D, G, J and P) indicate the regions of higher magnification in the right-adjacent images (B, E, H, K and Q)

Immunohistochemical examination of tumour tissues showed columnar epithelia on the superficial layer of the acini and papillae, and cuboidal epithelia deep in the acinar regions expressed CK18 and Pan-CK strongly throughout (Fig. 3A, B, G and H) and CK7 moderately throughout (Fig. 3D and E). Olfactory marker protein was not expressed in tumour epithelial cells (Fig. 3J and K). Kiel 67-positive nuclei were found in both tumour epithelial cells and stromal cells (Fig. 3M and N), resolving to a Kiel 67 index of approximately 23%.

### Ultrastructural characteristics

**Fig. 4.**
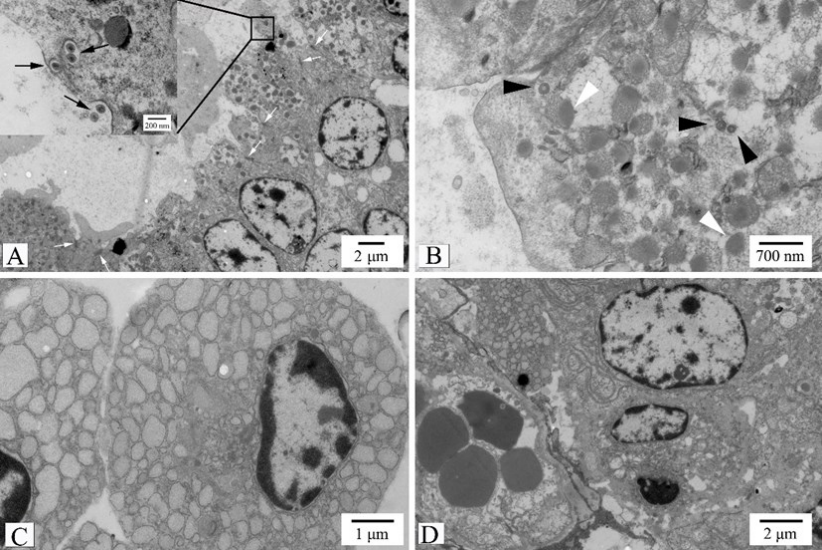
Ultrastructural manifestations of enzootic nasal adenocarcinoma. A – mature retrovirus-like particles in the intracytoplasmic vacuole and outside the cell (black arrows). Neighbouring tumour cells connected with tight junctions (white arrows). Scale bar = 2 μm. Corner view – details of the area indicated by the black box. Scale bar = 200 nm; B – immature intracytoplasmic particles (black arrowheads) and many secretory granules with heterogeneous density (white arrowheads) in the cytoplasm. Scale bar = 700 nm; C – sparse connections between stromal cells, and wide and obvious dilatation and vesiculation of the rough endoplasmic reticulum. Scale bar = 1 μm; D – small blood vessels in the stroma. Scale bar = 2 μm

Ultrastructurally, retrovirus-like particles 120 nm in diameter were found both inside and outside the tumour cells surrounding the acinar lumen (Fig. 4A). The mature extracellular particles were spherical, surrounded by a lipid envelope, and had a round but eccentrically positioned inner core. Mature *Betaretrovirus* particles in the intracytoplasmic vacuole had a fine structure (Fig. 4A corner diagram). The immature intracytoplasmic particles had no envelope and were close to the cell membrane (Fig. 4B).

The tumour cells were cuboidal in shape and were connected to each other by desmosomes and tight junctions (Fig. 4A). Short and sparse microvilli-like structures were observed on the cell surface. The nucleus had a round or oval shape and one or two nucleoli, with a predominance of euchromatin and without evident atypia (Fig. 4). There was an abundance of ribosomes and endoplasmic reticulum in the cytoplasm, and some rough endoplasmic reticula had wide and obvious dilatation and vesiculation (Fig. 4C). Moreover, in the cytoplasm near the acinar lumen, there were many secretory granules 200–700 nm in diameter. The secretory granules had an uneven electron density and a high density on one side, and resembled a bull’s eye in their shape (Fig. 4B). Small blood vessels and lymphocytes were also observed within the stroma (Fig. 4D).

## Discussion

Enzootic nasal tumour virus belongs to the *Retroviridae* family, which was divided into two subfamilies, *Orthoretrovirinae* and *Spumaretrovirinae*, in the 2023 report by the International Committee on Taxonomy of Viruses (19). This virus itself was not listed in the ICTV report. Gene sequence analysis shows similarity between ENTV and jaagsiekte sheep retrovirus in the *Betaretrovirus* genus (4). The Chinese reports of ENA in goats mainly focus on cases from Sichuan, Fujian and Guangdong provinces in the last five years (11). The goat farm in this study is located in Anhui province. An ENTV-2 strain isolated from Anhui province showed over 98% genome sequence homology with a strain isolated from Fujian province in a follow up study (6, 10). Since 2019, respiratory signs have been observed on a goat farm, confirmed as ENA through autopsy or PCR testing. The overall incidence rate ranged from 3% to 5%, and the affected goats ultimately died. No breed, gender, or age predisposition was found.

In the present study, histopathological examination revealed that ENA had a layered structure, with papillary formations in the superficial regions and acinar formations in the deeper tissue layers. These structural patterns represented distinct morphological features rather than separate histological subtypes. The composition of the papillae was similar to that of the original ethmoidal labyrinth mucosa, and the lymphoid follicles within the normal turbinate bone mucosa were retained. In contrast, the tumour cells located deep in the tumours were dominated by poorly differentiated nested clusters of cells, and the acinar lumens had almost disappeared. Periodic acid–Schiff staining of ENA also revealed that PAS-positive particles, which correlated with the mucus content, were mainly found in the cytoplasm of the acini, whereas they were almost absent in the cytoplasm of the papillae. Therefore, it can be speculated that the papillae on the tumour surface were formed by extrusion and hyperplasia of the turbinate mucosa during tumour growth. The tumour was highly differentiated at the beginning of the disease, and the hyperplastic, diseased acinus secreted a large amount of mucus. Subsequently, the tumour grew further, and the highly differentiated tumour cells were gradually replaced by poorly differentiated tumour cells.

Epithelial–mesenchymal transition is a biological process that converts epithelial cells into mesenchymal cells. In the study of epithelium-originating malignant tumours, EMT is a key process by which cancer cells acquire invasive and migratory properties. During this process, epithelial cells acquire characteristics of mesenchymal cells, such as enhanced motility and invasiveness (8). In this study, EMT was evident in the fibrous stroma of ENA tumour tissue. Although ENA is a low-grade adenocarcinoma with well-differentiated vasculature and few mitotic images (0 to 1 per 10 high-power fields), the marked EMT and bone destruction suggest that ENA may have malignant potential.

Pancytokeratin is a wide spectrum cytokeratin localised in the cytosol that is used to identify epithelial and nonepithelial cells. Cytokeratins 7 and 18 are low-molecular-weight cytokeratins that are also localised in the cytosol and are expressed in adenocarcinomas of epithelial origin (16). Cytokeratin 7 can label glandular tubular epithelia and is generally expressed in the epithelia of the respiratory tract and mammary gland (22). Cytokeratin 18 is primarily used as a positive marker for monolayer epithelia and is a negative marker in compound squamous epithelia, and therefore is often used in the differential diagnosis of squamous cell carcinoma and adenocarcinoma (12). Olfactory marker protein is localised to the cytosol of olfactory epithelial cells (1, 13). In this study, the immunohistochemical characteristics of the tumours indicated that ENA was adenocarcinoma, and tumour cells originated from the glandular tubular epithelium rather than the olfactory epithelium. The two keratins evaluated in this research can be considered immunophenotypes for identifying ENA tumour cells.

Kiel 67 is a type of nuclear protein that is expressed in the gap (G)1, synthesis, G2 and mitotis phases of the cellular cycle but not in the G0 phase. It is generally believed that the cells of more malignant tumours will proliferate more vigorously, grow faster and express more Kiel 67. Therefore, Kiel 67 has been widely used in tumour research to estimate malignancy and prognosis (7). This study shows that the Kiel 67 index of tumour stromal epithelial cells was approximately 23%, which is close to the lower limit of the index of highly aggressive tumours (30%). Another immunohistochemical study of ENA tumour tissues found that the Kiel 67 index was between 15% and 20% in advanced stages (14). All of these findings suggest that ENA has a potentially malignant capacity.

Electron microscopy showed the presence of enveloped virus particles outside the cells, as in some previous studies (3). In the present study, mature viral particles were found extracellularly, in the acinar lumen, and in intracytoplasmic vacuoles, and immature viral particles were also found in acinar tumour cells.

## Conclusion

This study is the first to explicitly demonstrate that ENA is a low-grade adenocarcinoma with malignant potential and proves that ENA tumour cells originated from the luminal epithelium of the nasal glands rather than the olfactory epithelium. Antibodies against CK7 and CK18 can be used as novel immunohistochemical identifiers of ENA tumour cells. Immature or mature retrovirus-like particles are present inside and outside the acinar cells.
